# Supplementary figures and images for: A multiplexed siRNA screen identifies key kinase signaling networks of brain glia
Source: Life Sci Alliance. 2023 Mar 6;6(5):e202201605. doi: 10.26508/lsa.202201605 (PMC9990460; doi:10.26508/lsa.202201605)

**Figure 6E**

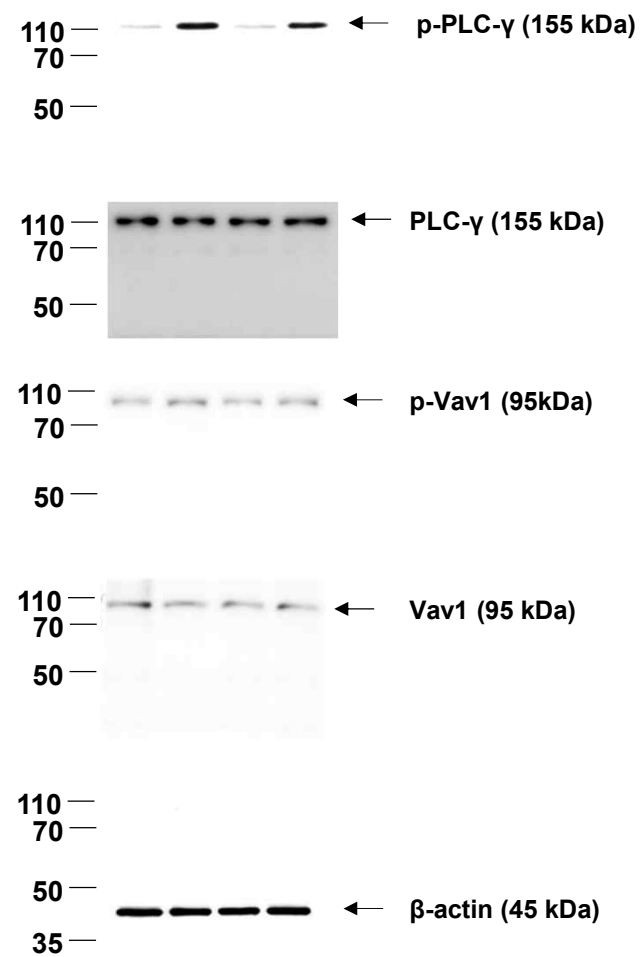

Supplement: Supplementary file 12 [file LSA-2022-01605_SdataF6.pdf]

**Figure 7B**

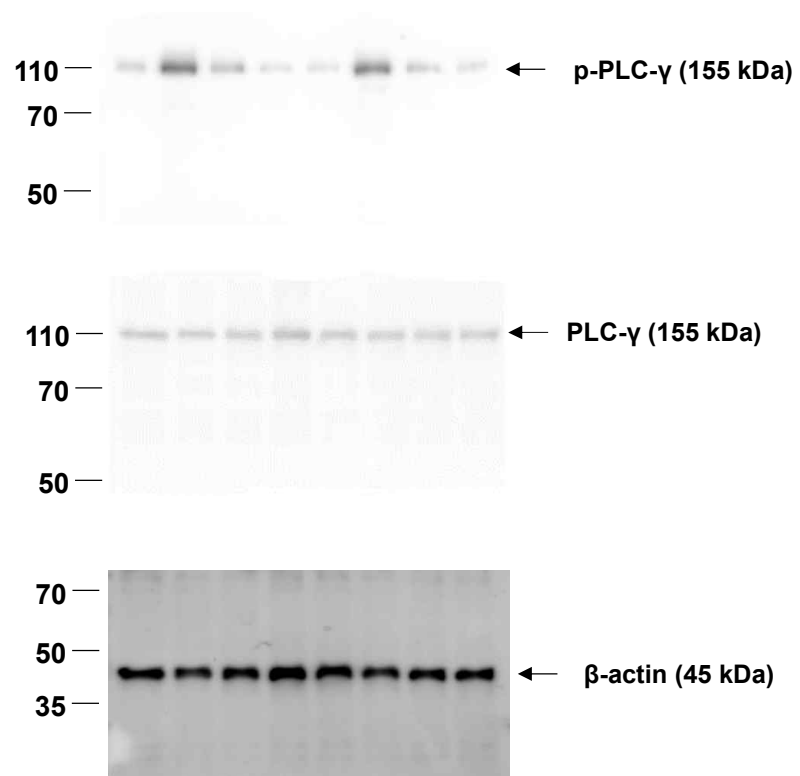

**Figure 7E**

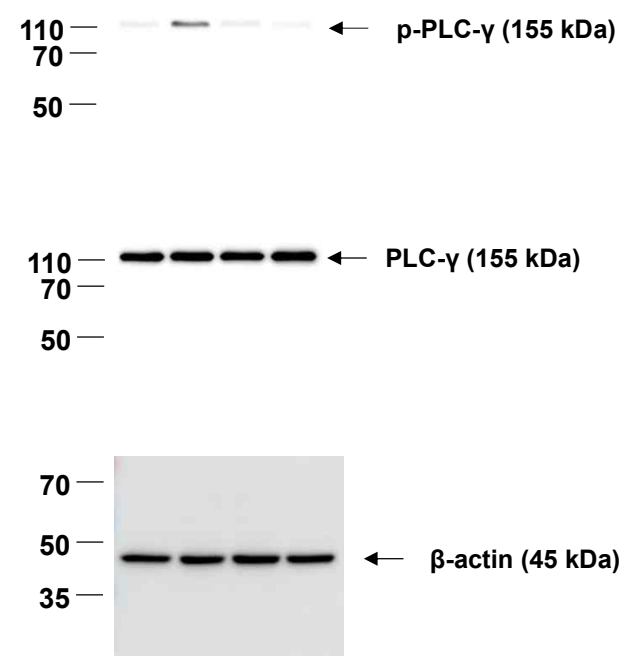

Supplement: Supplementary file 13 [file LSA-2022-01605_SdataF7.pdf]
